# Describing the minimally clinically important difference of a chemotherapy-induced peripheral neuropathy patient-reported outcome measure in young adults

**DOI:** 10.1016/j.apjon.2025.100656

**Published:** 2025-01-19

**Authors:** Robert Knoerl, Emanuele Mazzola, Lindsay Frazier, Roy L. Freeman, Marilyn Hammer, Ann LaCasce, Jennifer Ligibel, Marlise R. Luskin, Donna Berry

**Affiliations:** a Phyllis F. Cantor Center for Research in Nursing and Patient Care Services, Dana-Farber Cancer Institute, Boston, MA, USA; b Department of Data Science, Dana-Farber Cancer Institute, Boston, MA, USA; c Department of Pediatric Oncology, Dana-Farber Cancer Institute, Boston, MA, USA; d Department of Neurology, Beth Israel Deaconess Medical Center, Boston, MA, USA; e Department of Medical Oncology, Dana-Farber Cancer Institute, Boston, MA, USA; f Biobehavioral Nursing and Health Informatics, University of Washington, Seattle, WA, USA; g Department of Health Behavior and Clinical Sciences, University of Michigan School of Nursing, Ann Arbor, MI, USA

**Keywords:** Patient-reported outcome measures, Chemotherapy-induced peripheral neuropathy, Young adult, Neoplasms, Reliability, Validity

## Abstract

**Objective:**

The purpose of this secondary analysis was to characterize the reliability, validity, and minimally clinically important difference (MCID) of change scores over time of the European Organisation for Research and Treatment of Cancer Quality of Life Questionnaire-CIPN-20 item (QLQ-CIPN20) in young adults receiving paclitaxel or vincristine.

**Methods:**

Fifty young adults receiving vincristine or paclitaxel for the treatment of cancer completed the QLQ-CIPN20 at three time points associated with increasing cumulative chemotherapy dose. The Subject Significance Questionnaire was completed at T3. The analyses were focused on the calculation of floor and ceiling effects, internal consistency reliability, longitudinal validity, construct validity, and the MCID using an anchor-based approach for the QLQ-CIPN20 sensory and motor subscales.

**Results:**

By T3, 50% and 52% of participants reported QLQ-CIPN20 sensory and motor subscale scores at the floor, respectively. The internal consistency reliability of the sensory (*α* ​= ​0.83) and motor (*α* ​= ​0.89) subscales was strong. The Cohen’s *d* from T1 to T3 for the QLQ-CIPN20 sensory (*d* ​= ​−0.57) and motor (*d* ​= ​−0.47) subscales were small to moderate. There were low to moderate correlations between QLQ-CIPN20 sensory (*r* ​= ​0.45) and motor (*r* ​= ​0.27) subscale scores and vincristine cumulative dose. The MCID for worsening QLQ-CIPN20 sensory and motor subscale scores was 14.37 and 9.57, respectively (*P* ​< ​0.01).

**Conclusions:**

Study results provided preliminary evidence surrounding the MCID for worsening of QLQ-CIPN20 scores using an anchor based on young adults' perceived change in CIPN severity. Further research is needed to develop psychometrically sound CIPN patient-reported outcome measures to effectively evaluate the impact of CIPN interventions among young adults.

## Introduction

Cancer is the top illness-related cause of mortality in adolescents and young adults (15–39 years old).^1^ Neurotoxic chemotherapy regimens involving vincristine and paclitaxel are mainstays in the treatment of leukemia, Hodgkin/non-Hodgkin lymphoma, and/or breast cancer, some of the most common cancers experienced by young adults.^2^ Chemotherapy-induced peripheral neuropathy (CIPN) is a frequent consequence^3^ of neurotoxic chemotherapy administration and is characterized by sensory (e.g., paresthesia) and/or motor (e.g., weakness) changes in the hands and feet that may impair physical functioning.^4^ However, of the 38 clinical trials testing CIPN interventions since 2015,^5^ none have been conducted in young adults.

A barrier to the development of CIPN interventions for young adults is that little is known about optimal CIPN patient reported outcomes for this patient population.^6^ CIPN should be measured using patient-reported outcome and clinician-rated instruments in CIPN intervention trials.^7^ The Total Neuropathy Score (TNS©)^8^^,^^9^ is likely the best clinician-rated measure of CIPN in young adults because its' variants (e.g., Clinical Version) have demonstrated sufficient reliability and validity across the lifespan.^10^, ^11^, ^12^, ^13^, ^14^ One promising patient-reported outcome measure of CIPN is the Quality of Life Questionnaire-Chemotherapy-Induced Peripheral Neuropathy twenty-item scale (QLQ-CIPN20).^15^ The QLQ-CIPN20 has undergone extensive psychometric testing^16^, ^17^, ^18^ and is considered one of the best instruments for the measurement of CIPN in adults.^16^ Although, the reliability and validity of this measure has not been assessed in young adults to date. Further, little is known about the minimally clinically important difference (MCID) in QLQ-CIPN20 scores over time to determine what constitutes clinically meaningful changes in CIPN severity. Using a distribution-based approach, Yeo et al. (2019) determined the MCID for the QLQ-CIPN sensory & motor subscales in adults to be 2.5–5.9 and 2.6–5.0, respectively (*n* range ​= ​191–287). A limitation of the study was that there was a floor effect in QLQ-CIPN20 scores. As CIPN severity is dose dependent, the observed floor effects may have been attributed to suboptimal CIPN measurement timing (i.e., 3 weeks after chemotherapy initiation may not be a sufficient amount of time for CIPN to develop; 12 months after chemotherapy initiation may allow for natural reduction in CIPN severity).^19^

The identification of a reliable and valid CIPN patient-reported outcome measure for young adults will aid in the determination of efficacious CIPN interventions and sample size estimation in future trials.^20^ The purpose of this secondary analysis was to characterize the internal consistency reliability, construct validity, longitudinal validity, and MCID for change score scores over time of the QLQ-CIPN20 in young adults receiving paclitaxel or vincristine.

## Methods

### Sample and setting

The data for this secondary analysis were derived from a longitudinal, observational study designed to determine the association between plasma nicotinamide adenine dinucleotide (NAD+) levels and CIPN severity in young adults receiving vincristine or paclitaxel.^21^ The sample consisted of 50 English speaking young adults (18–39 years old) with breast cancer, leukemia, or lymphoma who were beginning vincristine or paclitaxel chemotherapy (i.e., no other neurotoxic agents such as platinums) at Dana-Farber Cancer Institute and did not have neuropathy. Study oversight was provided by the Dana-Farber/Harvard Cancer Center Office for Human Research Studies (19-862; no clinical trial registration). Verbal consent was obtained from all study participants due to the need for social distancing-related to the COVID-19 pandemic. The following describes the measures, procedures, and statistical analyses pertinent to this secondary analysis of the measurement properties of the QLQ-CIPN20 in young adults receiving vincristine or paclitaxel.

One important change was made to the conduct of this secondary analysis prior to the initiation of the trial. We were initially going to measure the concurrent validity of the QLQ-CIPN20 sensory and motor subscales by comparing the association between QLQ-CIPN20 sensory and motor subscale scores and TNS© – Clinical Version^8^^,^^9^ scores at the end of the study. However, we were unable to administer the TNS© - Clinical Version (i.e., an in-person clinical exam) due to the need for social distancing related to the COVID-19 pandemic.

### Measures

#### European organisation of research and treatment of cancer QLQ-CIPN20

Participants' scores on the QLQ-CIPN20 sensory and motor subscales were evaluated in the analyses. The nine-item sensory subscale consists of questions focused on participants' self-report of numbness, tingling, and pain (e.g., shooting or burning) severity in the hands or feet. The eight-item motor subscale consists of questions focused on participants' self-report of upper or lower extremity weakness or cramping, or difficulties with fine/gross motor tasks. Sensory and motor subscale scores are transformed to a 0–100 scale, with higher scores representing worse sensory or motor CIPN, respectively.^15^ Strong evidence supports the internal consistency reliability, concurrent validity, and divergent validity of the QLQ-CIPN20 in adults who received neurotoxic chemotherapy.^16^^,^^22^

#### Subject significance questionnaire

The Subject Significance Questionnaire (SSQ) was administered to aid in the determination of the MCID of the QLQ-CIPN20 sensory & motor subscales. The SSQ is a five-item scale that measures participants' perceived level of improvement or worsening pertaining to the five QLQ-C30 domains.^23^, ^24^, ^25^ We adapted the SSQ to create a two-item scale that asks participants to rate their perceived level of change related to the QLQ-CIPN20 sensory & motor subscales from their first neurotoxic chemotherapy infusion. Each item is scored from “very much worse” (1) to “very much improved” (7).

### Procedures

Young adults in the primary study completed the QLQ-CIPN20 sensory and motor subscales at three time points during paclitaxel or vincristine chemotherapy associated with increasing CIPN severity:^26^^,^^27^ T1 – prior to the first paclitaxel or vincristine infusion, T2: 3–5 mg vincristine or 350–450 mg/m^2^ paclitaxel, T3: 7–9 mg vincristine or 700–900 mg/m^2^ paclitaxel. The SSQ was completed at T3 only.

### Data analysis

QLQ-CIPN20 sensory and motor subscale scores were described and evaluated for floor/ceiling effects. Internal reliability of the QLQ-CIPN20 sensory & motor subscales was calculated using Cronbach’s Alpha. Construct validity was examined by comparing the change in QLQ-CIPN20 sensory & motor subscale scores with changes in cumulative paclitaxel or vincristine dose from T1 to T3 using Spearman’s correlation. A Cohen’s *d* effect size was calculated to quantify the QLQ-CIPN20 sensory & motor subscale scores' longitudinal validity from T1 to T3. To incorporate participant feedback on change in CIPN severity, we used an anchor-based approach to determine the MCID of the QLQ-CIPN20 sensory and motor subscales.^23^^,^^28^ Anchored on SSQ ratings indicating “improvement” (score ​= ​5–7), “the same” (score ​= ​4), or “worsening” (score ​= ​1–3),^28^ mean change scores from T1 to T3 were calculated for the QLQ-CIPN20 sensory & motor subscales. The MCID was determined as the mean difference in QLQ-CIPN20 sensory & motor score changes reflecting a one category change on SSQ rating, using linear regression.

## Results

### Sample characteristics

Data from 50 participants were available for inclusion in the secondary analyses. Participant flow through the study and demographic characteristics have been previously reported.^21^ Participants were recruited from October 2020 to September 2022. In summary, participants were an average of 35 years old and mainly female (88%), white (78%), and diagnosed with breast cancer (78%). Approximately 78% of participants received paclitaxel and 22% received vincristine.^21^

### Response distribution of the QLQ-CIPN20 sensory and motor subscale scores

**Fig. 1:**
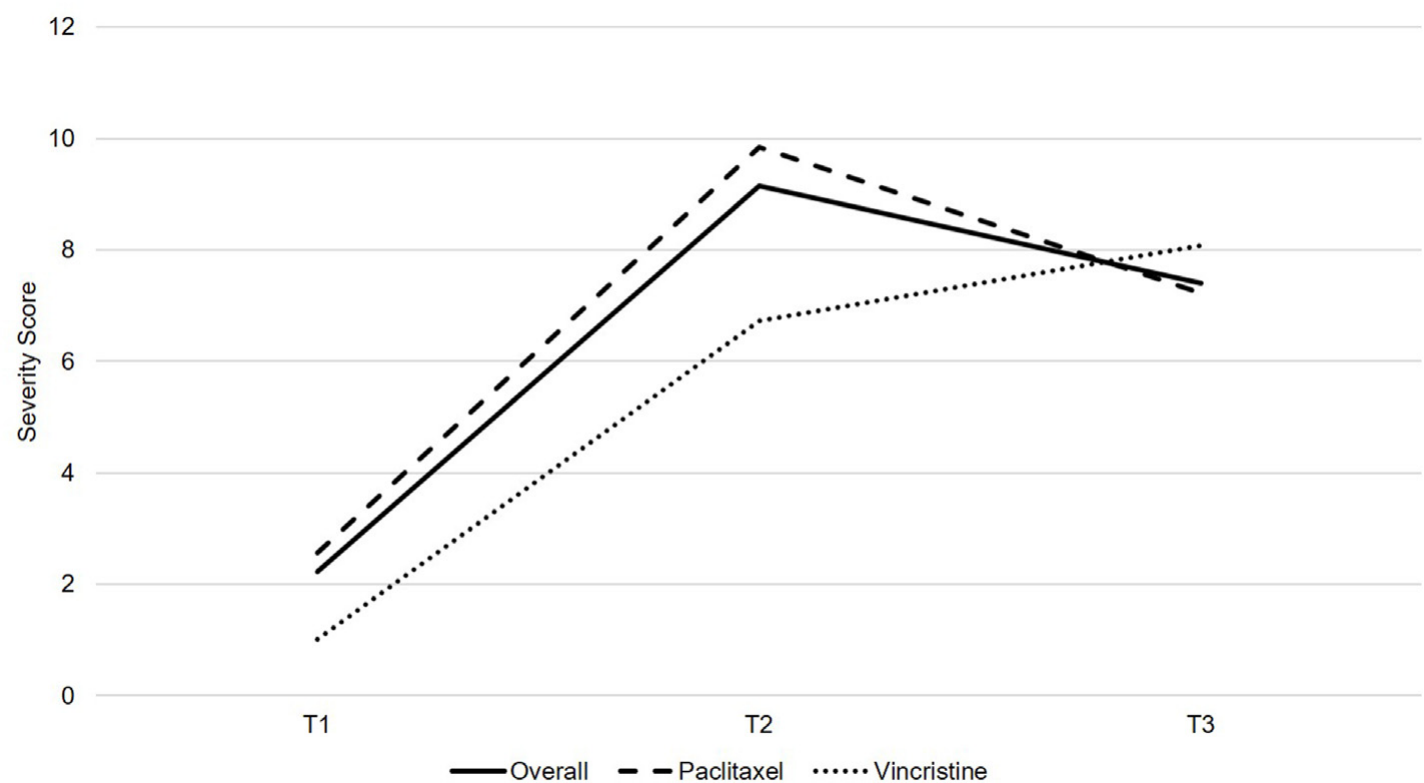
QLQ-CIPN20 sensory subscale scores among individuals receiving paclitaxel (*n* ​= ​39) or vincristine (*n* ​= ​11) from T1 to T3. QLQ-CIPN20, Quality of Life Questionnaire-Chemotherapy-Induced Peripheral Neuropathy twenty-item scale.

**Fig. 2:**
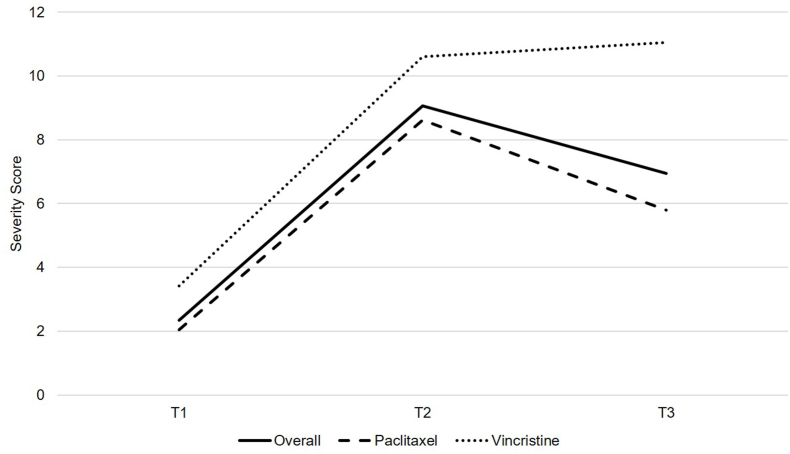
QLQ-CIPN20 motor subscale scores among individuals receiving paclitaxel (*n* ​= ​39) or vincristine (*n* ​= ​11) from T1 to T3. QLQ-CIPN20, Quality of Life Questionnaire-Chemotherapy-Induced Peripheral Neuropathy twenty-item scale.

Fig. 1, Fig. 2 describes the response distribution of QLQ-CIPN20 sensory and motor subscale scores from T1 to T3 among the overall sample, participants receiving paclitaxel (i.e., 80 mg/m^2^ weekly or 175 mg/m^2^ every 14 days), and participants receiving vincristine (i.e., 2 mg every seven or 21 days), respectively. There were a substantial number of scores at the floor for the sensory (50%) and motor (52%) subscales at T3 among the overall sample. There were no sensory or motor subscale scores at the ceiling at any time point. Overall, by T3, participants receiving vincristine (*n* ​= ​11) experienced worse sensory and motor neuropathy than participants receiving paclitaxel (*n* ​= ​39).

### Reliability, construct validity, and longitudinal validity

The Cronbach’s Alpha for the QLQ-CIPN20 sensory subscale was 0.83 and the Cronbach’s Alpha for the QLQ-CIPN20 motor subscale was 0.89. Among participants receiving paclitaxel (*n* ​= ​39), there were low correlations between cumulative dose and QLQ-CIPN20 sensory (*r* ​= ​−0.19) and motor subscale (*r* ​= ​−0.20) scores over time. Additionally, correlation coefficients were opposite of the expected direction (i.e., expected direction of the correlation coefficients was positive as QLQ-CIPN20 scores should increase as cumulative chemotherapy dose increases). Among participants receiving vincristine (*n* ​= ​11), there was a low-moderate correlation between cumulative dose and QLQ-CIPN20 sensory (*r* ​= ​0.45) and motor subscale (*r* ​= ​0.27) scores over time. The Cohen’s *d* for change in QLQ-CIPN20 sensory and motor scores from T1 to T3 were −0.57 and −0.47, respectively.

### Relationship between QLQ-CIPN20 sensory and motor subscale scores and SSQ ratings

For the QLQ-CIPN20 sensory subscale, 40%, 38%, and 22% reported no change, worsening, or improvement in sensory CIPN symptoms from T1 to T3 on the SSQ. For the QLQ-CIPN20 motor subscale, 62%, 16%, and 22% reported no change, worsening, or improvement in motor CIPN symptoms from T1 to T3 on the SSQ. Table 1 describes mean changes in QLQ-CIPN20 sensory and motor subscale scores by SSQ category (i.e., no change, worsening, or improvement) during paclitaxel or vincristine chemotherapy. The mean change in QLQ-CIPN20 sensory and motor subscale scores was 5.19 (*SD* ​= ​10.23) and 4.61 (*SD* ​= ​8.47), respectively (i.e., scores reflect worsening CIPN over time). For most SSQ categories, participants self-reported worsening QLQ-CIPN20 sensory or motor subscale change scores during chemotherapy.

**Table 1:** Mean Change (*SD*) for QLQ-CIPN20 Sensory and Motor Subscale Scores during Paclitaxel and/or Vincristine Chemotherapy among SSQ Categories.

QLQ-CIPN20 Subscale	Overall (*n* ​= ​50)	No Change^a^	Improvement^b^	Worsening^c^
Sensory	5.19 (10.23)	−1.11 (5.65)	2.69 (7.79)	13.25 (10.05)
Motor	4.61 (8.47)	2.04 (6.58)	6.76 (9.78)	11.61 (9.35)

Table 1 describes mean changes in QLQ-CIPN20 sensory and motor subscale scores among varying SSQ categories (i.e., No Change, Improvement, Worsening). Increasing scores represent worsening neuropathy severity. QLQ-CIPN20, Quality of Life Questionnaire-Chemotherapy-Induced Peripheral Neuropathy twenty-item scale; SSQ, Subject Significance Questionnaire.

a SSQ Score ​= ​4, *n* ​= ​20 sensory, *n* ​= ​31 motor.

b SSQ Score ​= ​5–7, *n* ​= ​11 sensory, *n* ​= ​11 motor.

c SSQ Score ​= ​1–3, *n* ​= ​19 sensory, *n* ​= ​8 motor.

Table 2 describes mean changes in QLQ-CIPN20 sensory and motor subscale scores with respect to change in SSQ category from “no change” to “worse,” or “no change” to “improvement,” respectively. The MCID for worsening QLQ-CIPN20 sensory and motor subscale scores was 14.37 and 9.57, respectively (*P* ​< ​0.01). For example, a one category change in SSQ categorization (e.g., no change to worsening), was associated with a 14.37 increase in mean QLQ-CIPN20 sensory subscale scores from T1 to T3.

**Table 2:** The Relationship between QLQ-CIPN Sensory and Motor Change Scores and the SSQ Rating Categories during Paclitaxel and/or Vincristine Chemotherapy.

QLQ-CIPN20 Subscale	Intercept	Slope – Improvement	Slope - Worse
Sensory	−1.11 (−4.63, 2.41)	3.81 (−2.1, 9.7)	14.37∗ (9.32, 19.41)
Motor	2.04 (−0.71, 4.79)	4.73 (−0.64, 10.10)	9.57∗ (3.50, 15.64)

Table 2 describes mean changes (95% CI) in QLQ-CIPN20 sensory and motor scores from T1 to T3 with respect to change in SSQ class from “no change” to “worse,” or “no change” to “improvement,” respectively. Increasing scores represent worsening neuropathy severity. QLQ-CIPN20, Quality of Life Questionnaire-Chemotherapy-Induced Peripheral Neuropathy twenty-item scale; SSQ, Subject Significance Questionnaire.

∗*P* ​< ​0.01.

## Discussion

The purpose of this analysis was to evaluate the psychometric properties and MCID of the QLQ-CIPN20 sensory and motor subscales among young adults receiving neurotoxic chemotherapy. The QLQ-CIPN20 sensory and motor subscales demonstrated strong internal consistency reliability values among young adults receiving neurotoxic chemotherapy.^29^ The study results are consistent with previous research revealing Cronbach Alpha coefficients of 0.87 and 0.83 for the sensory and motor subscales in a pooled sample of patients who had received neurotoxic chemotherapy (*n* ​= ​1155).^30^ Further, in terms of longitudinal validity, our prior research has demonstrated similar, but lower Cohen’s d values for the sensory (Cohen’s *d* ​= ​0.41) and motor subscales (Cohen’s *d* ​= ​0.38) among adults receiving neurotoxic chemotherapy.^31^

By T3, approximately 50% of participants reported scores at the floor of the scoring range for the QLQ-CIPN20 sensory and motor subscales, respectively. Evidence suggests that floor effects are present if at least 15% of the sample report scores at the lowest end of the scoring range.^29^ There was a higher incidence of scores at the floor of the scoring range for the sensory and motor subscales than in a comparable study in which approximately 24% and 39% of adults receiving neurotoxic chemotherapy reported scores at the floor of the scoring range for the QLQ-CIPN20 sensory and motor subscales during neurotoxic chemotherapy (e.g., paclitaxel, oxaliplatin, bortezomib).^31^ It is possible that there was a high frequency of scores at the floor of the scoring range among the sample at T3 because 58% of the sample completed neurotoxic chemotherapy treatment by the T3 time point.^32^ Although, floor effects have been reported for various other CIPN measures such as the Patient-Reported Outcomes version of the Common Terminology Criteria for Adverse Events numbness and tingling severity and interference items,^17^^,^^31^ the Common Terminology Criteria for Adverse Events grading scale,^17^ and single item numerical rating scales of CIPN.^17^^,^^31^ Further research is needed to identify CIPN measures that are sensitive to detecting changes in CIPN severity among young adults receiving neurotoxic chemotherapy for use in CIPN prevention clinical studies.^7^

The study results identified QLQ-CIPN20 sensory and motor subscale score changes associated with young adults' perceived worsening in sensory or motor CIPN severity. Using young adults' perceived change in sensory or motor CIPN severity as an anchor, the MCID scores for worsening QLQ-CIPN20 sensory and motor subscale scores were 14.37 and 9.57, respectively. The MCID values for improvement in QLQ-CIPN20 sensory and motor change scores are not clinically relevant as the values indicated CIPN worsening, which is consistent with the expected pattern of CIPN severity during neurotoxic chemotherapy treatment. The use of young adults' perceived change in CIPN severity as an anchor was a strength of the analysis, but similar to Yeo et al. (2019),^19^ the findings are limited because approximately 50% of the sample did not experience CIPN at the T3 time point despite that CIPN was measured at time points associated with increasing CIPN severity.^26^^,^^27^

The MCID values observed in this study are higher than findings observed in previous studies exploring MCID values for the QLQ-CIPN20.^19^^,^^33^ Most recently, Li et al. (2023) used anchor-based (i.e., grading per the Common Terminology Criteria for Adverse Events) and distribution based approaches (i.e., 0.5 *SD* of QLQ-CIPN20 score at each time point) to estimate the MCID for the QLQ-CIPN20 among adults receiving various neurotoxic agents (*n* ​= ​406).^33^ Results from the anchor-based analyses indicated that the minimally important difference for the QLQ-CIPN20 from baseline to end of treatment was 7.32, while for the same time points, the distribution-based analysis yielded a value of 5.52. There are several strengths to the findings reported by Li et al. (2023), including the large sample size, measurement of CIPN at baseline and end of treatment (i.e., ∼80% reported CIPN by end of treatment), and data supporting the Common Terminology Criteria for Adverse Events as an appropriate clinical anchor.^33^ It is possible that evaluating the MCID of the QLQ-CIPN20 as a whole is more appropriate since recent data has called into question the factor structure of the QLQ-CIPN20.^30^^,^^34^ On the other hand, other data recommends removing four (i.e., mainly consisting of items from the autonomic subscale) items of the twenty-item measure due to low item–item score correlations.^30^

Future work is needed to further verify MCID findings for the QLQ-CIPN20 and/or its subscales as the literature to date suggests that the MCID could range from 2.5^19^ to 14.37 depending on the MCID approach implemented and QLQ-CIPN20 derivation tested. In addition, further research should be directed towards calculating MCID values for QLQ-CIPN20-related improvements in CIPN severity. Studies to date have evaluated the MCID of the QLQ-CIPN20 when following participants during neurotoxic chemotherapy.^19^^,^^33^ Such designs increase the difficulty of calculating MCID values for improvement as CIPN severity is expected to increase during neurotoxic chemotherapy treatment without intervention. The identification of MCID values for improvement may be used to determine the clinical significance of pharmacological or non-pharmacological interventions for CIPN management in clinical studies.

The MCID and psychometric findings have limited implications for practice as the QLQ-CIPN20 is lengthy and complicated to score (e.g., requires score transformations) and due to the small sample size included in the analysis. Nevertheless, the results from the construct validity analyses, in addition with supporting data from the literature, raise the question as to whether dose reduction is an effective strategy to mitigate CIPN during chemotherapy treatment.^35^ While increasing cumulative dose of neurotoxic chemotherapy is recognized as a primary risk factor of CIPN incidence or severity,^36^ the study results demonstrated low to moderate correlations between sensory or motor CIPN severity and increasing paclitaxel or vincristine cumulative dose over time. Previous reports have demonstrated similar lack of associations between CIPN severity and neurotoxic chemotherapy cumulative dose.^35^^,^^37^ Additionally, evidence demonstrates that women with breast cancer who received dose reduction during paclitaxel treatment experienced greater self-reported CIPN severity approximately three months after treatment completion in comparison to women who did not receive a dose reduction.^27^ The present study was not aimed to determine the utility of dose reduction as a management strategy for worsening CIPN during neurotoxic chemotherapy, but the emerging data from the literature suggest that this question should be prioritized in future research given the negative effects dose reduction may pose for cancer treatment.

### Limitations

There are several limitations to this research. First, the study results may not be generalizable to adolescents as no adolescents (i.e., 15–17 years old) were enrolled. Similarly, the enrolled sample mainly consisted of white, non-Hispanic, females with breast cancer receiving paclitaxel that were recruited from one institution, thereby limiting the external generalizability of the study results. Second, the findings are exploratory as the statistical analyses were likely underpowered and participants were receiving vincristine or paclitaxel with varying delivery schedules. Third, CIPN severity, as quantified by the QLQ-CIPN20, was low among young adults. The low CIPN severity may have hindered our ability to accurately explore the psychometric properties of the measure because most scores were skewed toward no neuropathy severity.

### Conclusions

Study results provided preliminary evidence surrounding the MCID for worsening of QLQ-CIPN20 sensory and motor subscale scores using an anchor based on young adults' perceived change in CIPN severity. Given the psychometric weaknesses identified with the QLQ-CIPN20, further research is needed to further develop psychometrically sound CIPN patient-reported outcome measures to effectively evaluate the impact of novel CIPN interventions among young adults.

## **CRediT authorship contribution statement**

RK: conceptualization, methodology, investigation, writing – original draft, funding acquisition, project administration. DB and MH: supervision, writing – review & editing, conceptualization. EM: formal analysis, conceptualization, writing – review & editing. LF, RLF, AL, JL, and MRL: conceptualization, writing – review & editing. All authors had full access to all the data in the study, and the corresponding author had final responsibility for the decision to submit for publication. The corresponding author attests that all listed authors meet authorship criteria and that no others meeting the criteria have been omitted.

## **Ethics statement**

Study oversight and institutional review board approval was provided by the Dana-Farber/Harvard Cancer Center Office for Human Research Studies (19-862). Verbal informed consent was obtained from all study participants. A waiver of documentation of informed consent was approved by the institutional review board due to the minimal risk nature of the study and the need for social distancing due to the COVID-19 pandemic.

## Funding

Research reported in this publication was supported by the 10.13039/100000056National Institute of Nursing Research of the National Institutes of Health under Award Number K23NR018689. The content is solely the responsibility of the authors and does not necessarily represent the official views of the National Institutes of Health. The funders had no role in considering the study design or in the collection, analysis, interpretation of data, writing of the report, or decision to submit the article for publication.

## **Declaration of competing interest**

**RK** has received personal fees from the Comprehensive and Integrative Medicine Institute. **ML** has received research funding from 10.13039/100004336Novartis and Abbive and serves on advisory boards for 10.13039/100004319Pfizer, 10.13039/100004336Novartis, and Jazz. The corresponding author, RK, serves as a member of the editorial board of the *Asia-Pacific Journal of Oncology Nursing*. The article has undergone the journal's standard publication procedures. No other authors have any disclosures to report.

## **Data availability statement**

The data used and/or analyzed during the current study are available from the corresponding author, RK, on reasonable request.

## Declaration of generative AI and AI-assisted technologies in the writing process

No AI tools/services were used during the preparation of this work.
